# Oral Probenecid for Nonhospitalized Adults with Symptomatic Mild-to-Moderate COVID-19

**DOI:** 10.3390/v15071508

**Published:** 2023-07-06

**Authors:** David E. Martin, Neelam Pandey, Purvi Chavda, Gurpreet Singh, Rakesh Sutariya, Frederic Sancilio, Ralph A. Tripp

**Affiliations:** 1 TrippBio, Inc., Jacksonville, FL 32256, USA; 2 PCMC’s PGI Yashwantrao Chavan Memorial Hospital, Pune 411018, India; 3 Zenovel Pharma Services LLP, Ahmedabad 380060, India; 4 Clearway Global, Stuart, FL 34997, USA; 5 Department of Infectious Diseases, College of Veterinary Medicine, University of Georgia, Athens, GA 30602, USA; ratripp@uga.edu

**Keywords:** COVID-19, viral dynamics, probenecid, antiviral, therapeutic

## Abstract

Probenecid is an orally bioavailable, uricosuric agent that was first approved in 1951 for the treatment of gout, but was later found to have potent, broad-spectrum antiviral activity against several respiratory viruses including SARS-CoV-2. We conducted a phase 2 randomized, placebo-controlled, single-blind, dose-range finding study in non-hospitalized patients with symptomatic, mild-to-moderate COVID-19. Patients were randomly assigned in a 1:1:1 ratio to receive either 500 mg of probenecid, 1000 mg of probenecid, or a matching placebo every 12 h for five days. The patients’ COVID-19 viral load hospitalization, or death from any cause through day 28, as well as safety, were evaluated. COVID-19-related symptoms were assessed at baseline, and on days 3, 5, 10, 15, and 28. The primary endpoints of the study were time to first negative SARS-CoV-2 viral test (or viral clearance) and the proportion of patients that were symptom-free at day 5. A total of 75 patients were randomized, with 25 patients in each group. All of the patients completed the study as planned with no hospitalizations or deaths being reported. The median time to viral clearance was significantly shorter for the probenecid 1000 mg group than for placebo (7 days vs. 11 days, respectively; *p* < 0.0001), and for the probenecid 500 mg group versus placebo (9 days vs. 11 days, respectively; *p* < 0.0001). In addition, the median time to viral clearance was significantly shorter for the probenecid 1000 mg group than for the probenecid 500 mg group (7 days vs. 9 days, respectively; *p* < 0.0001). All patients reported at least one COVID-19-related symptom on days 3 and 5; however, on day 10, a significantly greater proportion of patients receiving probenecid 1000 mg reported the complete resolution of symptoms versus placebo (68% vs. 20%, respectively; *p* = 0.0006), as well as for those receiving probenecid 500 mg versus placebo (56% vs. 20%, respectively, *p* = 0.0087). The incidence of adverse events during treatment was similar across all groups for any adverse event, and was 12%. All events were mild with no serious adverse events reported and no discontinuations due to an adverse event. The treatment of patients with symptomatic, mild-to-moderate COVID-19 with probenecid resulted in a significant, dose-dependent decrease in the time to viral clearance and a significantly higher proportion of patients reporting complete symptom resolution by day 10. (Supported by TrippBio; ClinicalTrials.gov number, NCT05442983 and Clinical Trials Registry India number CTRI/2022/07/043726).

## 1. Introduction

Infection with severe acute respiratory syndrome coronavirus 2 (SARS-CoV-2) resulting in coronavirus disease 2019 (COVID-19) was first reported in 2019 [1]. Since then, the global pandemic has resulted in >664,800,000 confirmed cases of COVID-19, with >6,700,000 deaths reported to the WHO [2]. The pandemic has led to an unprecedented medical and scientific response, including the development of multiple vaccines and several therapeutics which have received regulatory approval [3]. The world is now entering the endemic phase of the crisis with an ever-changing pandemic virus, as evidenced by the fact that many of the early antibody treatments and antiviral drugs are no longer effective [4] and that the ancestral mRNA vaccines are less immunologically effective [5]. While widespread drug resistance has not yet been reported, there is evidence of developing resistance, specifically to low doses of remdesivir [6].

The evolving nature of SARS-CoV-2 variants emphasizes the need to identify drug treatments that are safe, effective, and refractory to developing drug resistance. Antiviral drugs either target the virus or cellular components which are required for virus replication. Directly targeting the virus can lead to a narrow spectrum of antiviral activity and the likelihood of developing viral mutations and drug resistance. In contrast, antiviral drugs that target the host cell pathways that are usurped by viruses for replication are more likely to be broad-spectrum and inhibit the replication of multiple viruses. Traditional drug discovery pipelines are time- and resource-intensive but bridging high-throughput screening (HTS) with host gene silencing makes it possible to discover host pathways which are used by viruses for their replication [7]. Using RNA interference (RNAi) to screen human A549 cells infected with A/WSN/33 and other influenza virus strains has shown that the organic anion transporter-3 gene (OAT3), a member of the solute carrier (SLC) 22 family (SLC22), is an important host gene required for influenza virus replication [8]. As OAT3 is required for influenza virus replication, probenecid—a prototypic OAT3 inhibitor (IC_50_ = 1.25 µM)—was examined for antiviral activity. Probenecid was found to have potent in vitro inhibition against several influenza A strains and reduced lung titers in a mouse model [8]. More recent studies have shown that probenecid potently inhibits SARS-CoV-2 with an in vitro IC_50_ of 13 nM [9]. Studies in a Syrian hamster model of SARS-CoV-2 infection showed that probenecid, administered either prophylactically or post-infection, significantly reduces the lung titers of SARS-CoV-2 by approximately 4–5 logs [9]. In addition to the antiviral activity, probenecid has been shown to diminish NLRP3 inflammasome-dependent IL-1b secretion by macrophages, and probenecid intranasal treatment of mice reduced influenza disease associated with pro-inflammatory cytokine production and cellular infiltrates in the lung, and provided protection against disease [10]; further, probenecid has also been shown to be an inhibitor of PANX1 [11], which mediates the activation of caspase-1 and the release of IL-1β induced by P2X7 receptor activation [12], as well as modulating the expression of ACE2 [13].

Probenecid was initially developed to decrease the renal clearance of penicillin and increase plasma concentrations [14] but was later found to be effective at increasing the renal excretion of urate, decreasing serum uric acid levels, and improving symptoms in patients with gout [15]. It was first approved for use by the FDA in 1951 for the treatment of gout and is generally safe and well tolerated with no black box warnings when used at doses up to 2000 mg per day [16]. In order to select an appropriate dosing regimen, at or below the maximum FDA approved daily dose 2000 mg/day, a population pharmacokinetic (PK) model was developed using a one-compartment model with first-order absorption, lag time, and saturation metabolism [9]. The results of that analysis confirmed that doses administered twice daily would be expected to deliver plasma concentrations of probenecid that exceeded the protein binding-adjusted IC_90_ concentration for the entire dosing regimen to a greater extent than once-daily dosing. The top dose was, therefore, set at 1000 mg twice daily (b.i.d. for a total daily dose of 2000 mg) with the lower dose set at 500 mg b.i.d. to provide non-overlapping plasma concentrations for inclusion in a randomized, single-center, single-blind, placebo-controlled study to evaluate the efficacy of probenecid for the treatment of symptomatic, non-hospitalized patients with COVID-19 infection.

## 2. Methods

### 2.1. Trial Design and Oversight

This phase 2, dose-range finding study utilized a randomized, single-blind, and placebo-controlled design to evaluate the antiviral activity, clinical efficacy, and safety of probenecid orally administered, 500 mg or 1000 mg b.i.d., versus placebo in non-hospitalized, symptomatic patients with PCR-confirmed SARS-CoV-2 infection. Patients were enrolled in a single center at PCMCs PGI Yashwantrao Chavan Memorial Hospital in Pune, India.

The study was conducted in accordance with Good Clinical Practice guidelines and was approved by the local ethics committee (Skinovate Independent Ethics Committee, Pune, India). Written informed consent was obtained from all of the participants. The trial was designed by representatives of the sponsor. Safety oversight was performed by the sponsor and contract research organization (CRO). Data were collected by the investigator and site personnel, analyzed by statisticians employed by the CRO, engaged by the sponsor, and interpreted by the authors.

### 2.2. Patient Selection

Non-hospitalized adults aged 18–65 years with symptomatic, mild, or moderate COVID-19 were eligible. Mild or moderate illness was determined based on definitions adapted from World Health Organization (WHO) guidelines [17]. Patients within 5 days or less of randomization had to present with at least one early-onset COVID-19 symptom (i.e., fever or chills, cough, shortness of breath or difficulty breathing, fatigue, muscle or body aches, headache, a loss of taste or smell, sore throat, congestion or runny nose, nausea, vomiting or diarrhea) with laboratory confirmation of SARS-CoV-2 infection no more than 48 h prior to randomization. Key exclusion criteria were severe COVID-19 (either outpatient or hospitalized) or Long COVID-19 syndrome. Detailed eligibility criteria are listed in the protocol, available from the corresponding author.

### 2.3. Study Procedures

Patients were randomly assigned 1:1:1 to receive either probenecid 500 mg or 1000 mg b.i.d., or matching placebo b.i.d. for five days. Patients were blinded to study drug assignment while study personnel were unblinded. Patients were seen in the clinic on days 0 (screening visit), 1, 3, 5, 10, 15, and 28 (end-of-study visit). Safety and laboratory tests (hematology and biochemistry) were performed on days 1, 5, and 28, with a complete physical examination on days 0 and 28. Vital signs assessments, i.e., body temperature, blood pressure, oxygen saturation, respiratory rate, and heart rate, were established according to the WHO clinical progression scale [18], and nasal swabs were collected daily for quantitative SARS-CoV-2 RNA testing, i.e., from days 0 to 28 or until the patient tested negative. COVID-19-related symptoms were assessed during clinic visits at baseline on days 3, 5, 10, and 15, and at the end of the study.

### 2.4. Statistical Analysis

The primary endpoints of the study were: (1) time to virus RNA clearance as measured by quantitative RT-PCR analysis of nasopharyngeal swabs using a validated, laboratory-developed test based on the U.S. CDC 2019-nCoV Emergency Use Authorization assay, and (2) the proportion of patients with symptoms resolution at day 5 from baseline. Secondary endpoints evaluated the antiviral activity, symptom resolution, changes in the WHO clinical progression scale, and emergence of adverse events at each of the clinic visits along with the pharmacokinetics of probenecid.

Time to virus RNA clearance was estimated using the Kaplan–Meier (KM) method. The median time to virus RNA clearance along with the 95% confidence intervals is given for each treatment arm. The number and percentage of subjects with time to virus RNA clearance and who are censored are presented, as are the KM product-limit estimates of the 25th, 50th (median), and 75th percentiles with associated CIs (where estimable). Viral loads below the limit of detection of 250 copies/mL were imputed as 125 copies/mL.

The proportion of patients with symptom resolution at days 3, 5, 10, and 15 was assessed using chi-square statistics. The chi-square test was used for the comparison of proportions from all treatment groups. In addition to a *p*-value of the test, two-sided 95% confidence limits for the difference in response rates between the treatment groups was calculated.

The change in the WHO progressions scale at days 3, 5, 10, 15, and 28 was analyzed using ANCOVA. ANCOVA analysis was performed for change from baseline analysis. ANCOVA model with change from baseline as the response variable and the baseline value of respective parameter and treatment as independent variables was fit to test mean difference between the treatments in change from baseline. Adjusted least square (LS) mean and the standard error values along with the *p*-value and the 95% confidence interval of the difference are presented.

In the absence of previous efficacy data for probenecid, a sample size of 75 patients (25 patients/treatment regimen) was used.

A complete listing of all secondary endpoints is included in the protocol, available from the corresponding author.

### 2.5. Role of the Funding Source

This work was funded by TrippBio, Inc. (Florida, USA), which was involved in the study design, data collection, data analysis, data interpretation, and writing of this report. Probenecid 500 mg tablets were provided by GENO Pharmaceuticals Ltd., Goa, India and the matching placebo tablets were provided by Sushen Medicamentos Pvt. Ltd., Ahmedabad, India.

## 3. Results

### 3.1. Patient Demographics and Clinical Characteristics

A total of 75 patients were enrolled and completed the study as planned. The first patient was enrolled on 18 July 2022, and the last patient was enrolled on 9 September 2022. The patients were equally distributed across all of the treatment groups so that the groups had similar demographic characteristics (Table 1). Most of the patients had received at least one dose of either Covishield or Covaxin. Co-morbidities were uncommon, with hypertension and diabetes mellitus reported in <25% of patients.

### 3.2. Time to Viral Clearance and Viral Dynamics

**Figure 1:**
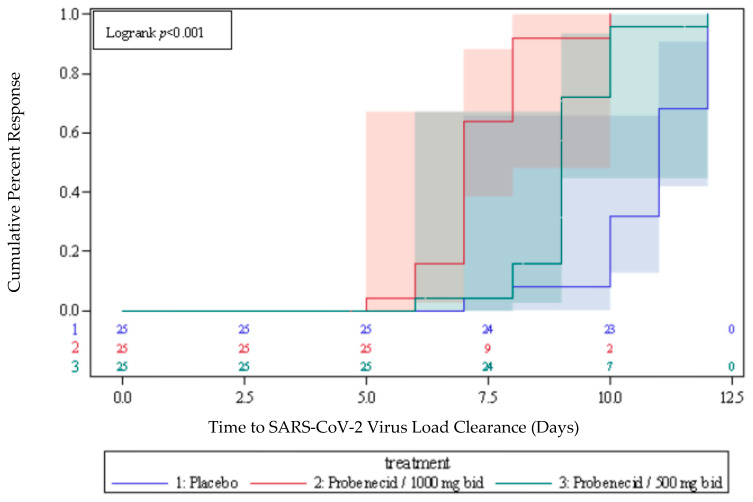
Time to viral load clearance. Shown is a Kaplan–Meier plot with 95% Hall–Wellner bands of time to clearance of SARS-CoV-2 RNA by treatment. Quartiles are derived from Kaplan–Meier product limit estimates. *P*-value is based on the Log-Rank test for comparing between treatment groups.

The time to clearance of virus RNA in nasopharyngeal swabs (<250 copies/mL) was the primary endpoint of this study and was significantly shorter for the probenecid 1000 mg group versus placebo (median of 7 days vs. 11 days, respectively; log-rank *p* < 0.0001), and for the probenecid 500 mg group versus placebo (median of 9 days vs. 11 days, respectively; log-rank *p* < 0.0001), as shown in Figure 1. The median time to viral clearance was significantly shorter for probenecid 1000 mg than for probenecid 500 mg (7 days vs. 9 days, respectively; log-rank *p* < 0.0001), as shown in Figure 1. All of the patients receiving probenecid 1000 mg cleared the virus by day 10 versus day 12 for probenecid 500 mg and placebo-treated patients.

**Figure 2:**
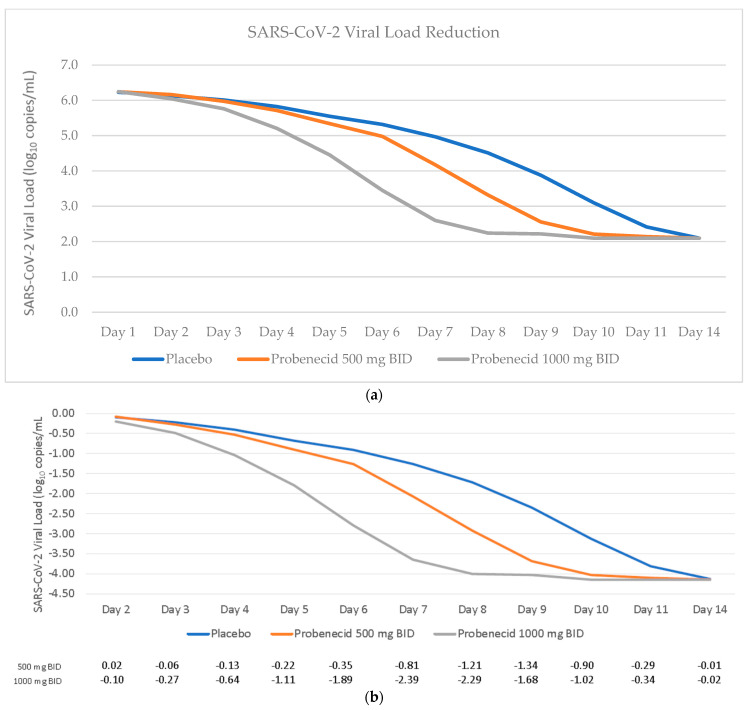
SARS-CoV-2 viral dynamics. Shown in top panel (**a**) is the mean SARS-CoV-2 viral load reduction (log_10_ copies/mL) over time by treatment: placebo (blue), probenecid 1000 mg b.i.d. (gray), and probenecid 500 mg b.i.d. (orange). Shown in the lower panel (**b**) is the least squares mean change from baseline in SARS-CoV-2 RNA (log10 copies/mL) by treatment: placebo (blue), probenecid 1000 mg b.i.d. (gray), and probenecid 500 mg b.i.d. (orange).

There was a marked effect of probenecid treatment on SARS-CoV-2 viral dynamics, with a dose-dependent decrease in viral load versus placebo throughout the sampling window (Figure 2a,b). The maximum difference between placebo and the probenecid 1000 mg treatment occurred on day 7, with an adjusted mean difference of −2.39 log10 copies/mL. The maximum difference from placebo for the probenecid 500 mg was at day 9, with an adjusted mean difference of −1.34 log10 copies/mL.

### 3.3. Time to Symptom Resolution

**Figure 3:**
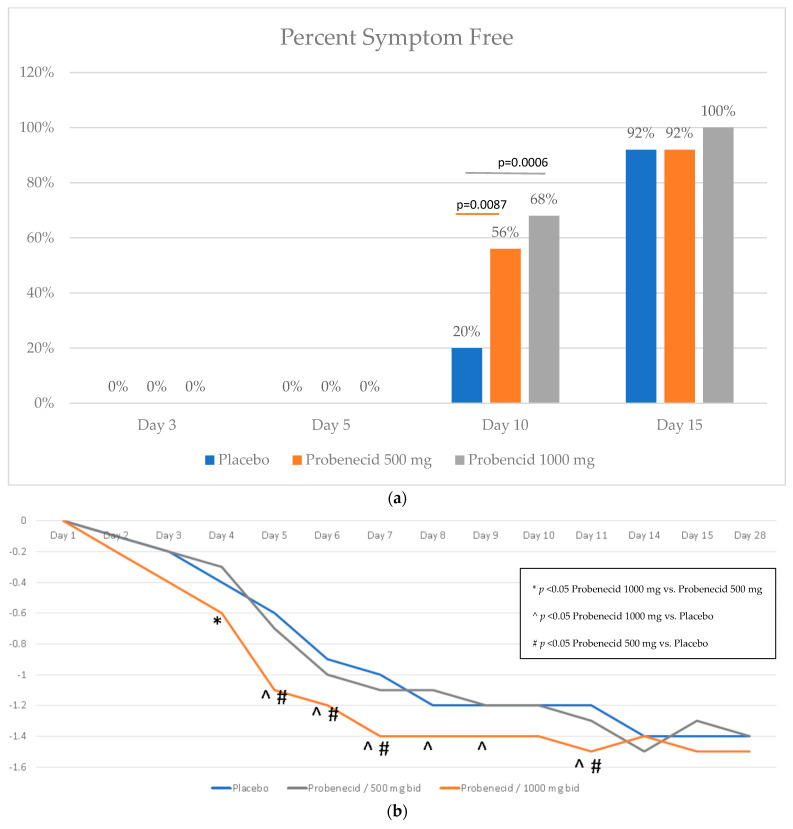
(**a**) Symptom resolution. Shown is the proportion of patients with complete symptom resolution at day 10 and day 15 by treatment: placebo (blue), probenecid 1000 mg b.i.d. (orange), and probenecid 500 mg b.i.d. (gray). (**b**) Temperature. the change from baseline for body temperature (°Celsius) over time for placebo (blue), probenecid 1000 mg b.i.d. (orange), and probenecid 500 mg b.i.d. (gray).

All of the patients with symptoms present at baseline reported symptoms on day 3 and day 5. At day 10, a significantly higher proportion of the patients receiving probenecid 1000 mg had resolved symptoms versus placebo (68%, [95% CI 46.5–85.1%] vs. 20%, [95% CI 6.8–40.7%]; log-rank *p* = 0.0006). In addition, a significantly higher proportion of the patients receiving probenecid 500 mg achieved symptom resolution versus placebo (56%, [95% CI 34.9–75.6%] vs. 20%, [95% CI 6.8–40.7%]; log-rank *p* = 0.0087). The symptom resolution difference between probenecid dose levels was not significantly different. All of the patients receiving probenecid 1000 mg had all of their symptoms completely resolved by day 15, with all of the patients in the probenecid 500 mg and placebo-treated groups reporting the complete resolution of symptoms by day 18. Data are shown in Figure 3a.

Fever was a prominent COVID-19-related symptom, and an exploratory analysis found a statistically significant difference in the change from baseline for the probenecid 1000 mg group versus either the placebo or probenecid 500 mg-dose groups (Figure 3b).

### 3.4. Disease Progression

All of the patients recorded a WHO Clinical Progression Scale score of 2 at the study entry (Table 2). Consistent with the findings of symptom reduction, there were no significant changes from baseline on study days 3 or 5. However, by study day 10, there was a significant difference in score between both treatment arms and placebo (Table 2). Importantly, no patients showed a worsening of symptoms, were hospitalized, or died during the study.

### 3.5. Safety

Probenecid was generally safe and well tolerated, with only mild adverse events reported (Table 3). The most frequent adverse events were gastrointestinal with nausea and vomiting reported in 8% (2/25) of patients receiving probenecid 1000 mg. No patient discontinued or interrupted treatment for any adverse event. No Grade 2 or higher laboratory abnormalities were noted, and all of the patients completed their dosing regimen.

## 4. Discussion

In this phase 2 dose-range finding study, probenecid was associated with a potent, dose-dependent antiviral effect and a significant reduction in COVID-19-related symptoms in non-hospitalized patients who were mildly to moderately symptomatic with COVID-19. Considerable antiviral activity was observed, with a significant reduction in the time required to reach an undetectable SARS-CoV-2 viral load and a significant difference in the change from baseline in SARS-CoV-2 viral RNA concentrations. There was a significant reduction in all COVID-19-related symptoms associated with probenecid versus placebo treatment.

Treatment with probenecid was generally safe and well tolerated, with only mild adverse events noted. Probenecid has been marketed since 1951 and has been used safely by millions of patients across the globe [17]. The adverse events noted in this study are similar to what is reported in the FDA-approved package insert [19].

The significance of OAT3 in viral replication was previously shown by the probenecid treatment of influenza, RSV, and SARS-CoV-2 infections [20]. Since the effects of probenecid appear to be pan-antiviral for these important respiratory viruses, the mechanism of action is likely enabled through a common host pathway. Various host genes have been shown to be important in virus replication. For RSV replication, importin β-1, Crm1, caveolin, and c-Jun N-terminal kinase (JNK) activity have been shown to be required [21,22]. Probenecid has been shown to inhibit JNK phosphorylation [23], and JNKs are among the most crucial mitogen-activated protein kinases (MAPKs) that regulate various cellular processes [24]. Typically, virus–receptor interaction initiates the MAPK signaling cascade, leading to JNK phosphorylation [25]. Activated JNK is translocated to the nucleus, activating specific transcription factors that result in altered gene expression [26]. It has been shown that hepatocyte nuclear factor -4 (hfn-4) regulates the expression of drug transporters by binding to the DNA of specific genes such as OATs [27]. Thus, probenecid inhibits JNK phosphorylation and downstream Hfn-4 expression, which regulates OAT3 gene expression, preventing virus assembly and replication. Therefore, JNK signaling leads to a pro-viral state, whereas probenecid treatment inhibits JNK signaling, favoring an anti-viral state. Importantly, probenecid also inhibits reactive oxygen species’ (ROS) generation by inhibiting the COX-2 and JNK pathways, which have been implicated in a variety of inflammatory responses [23], suggesting a dual antiviral and anti-inflammatory role for probenecid treatment.

Probenecid is relatively easy to manufacture and transport and has a long shelf-life [17]. These characteristics make probenecid an ideal candidate for use in lower-income countries that might not be able to access the necessary quantities of the currently available COVID-19 therapeutics.

There are limitations to this clinical study. Specifically, the study participants were all enrolled in a single center in India, and viral genetic assessments were not performed. However, published reports indicated that the prevalent circulating SARS-CoV-2 variant at the time of enrollment in the area was the Omicron variant BA.2.75 (23.03%), followed by BA.2.38 (21.01%), BA.5 (9.70%), BA.2 (9.09%), BA.2.74 (8.89%), and BA.2.76 (5.56%) [28]. The study was conducted in a single-blind manner with patients blinded to study treatment assignment. Although the study personnel were unblinded, every effort was made to ensure that the personnel interacting with the patients were unaware of treatment assignment.

In conclusion, treatment with probenecid was associated with a significant, dose-related decrease in the time to viral clearance and a significant increase in the proportion of patients with complete symptom resolution by day 10. Both dose levels of probenecid which were tested were generally safe and well tolerated. Based on these results, probenecid 1000 mg b.i.d. for five days will be further evaluated to characterize its efficacy in patients with COVID-19. Future studies will explore the efficacy of probenecid in more severely ill patients as well as those > 65 years of age.

## Figures and Tables

**Table 1:** Demographics and clinical characteristics at baseline.

	Probenecid 1000 mg	Probenecid 500 mg	Placebo
	*N* = 25	*N* = 25	*N* = 25
Age, median (range) years	39.1 (23–65)	40.5 (24–64)	44.0 (22–64)
Sex			
Male	15	19	18
Female	10	6	7
Race			
Asian	25	25	24
Black or African American			1
BMI, median (range) kg/m^2^	24.8 (20.0–30.5)	25.3 (19.3–33.2)	25.3 (20.2–30.0)
Co-Morbidities			
Diabetes Mellitus (%)	2 (8%)	2 (8%)	5 (20%)
Hypertension	4 (16%)	6 (24%)	6 (24%)
Baseline SARS-CoV-2 RNA, median (range) log_10_ copies/mL	6.3 (6.00–6.48)	6.3 (6.01–6.54)	6.2 (5.95–6.55)
Vaccine Status			
Y	17 (68%)	17 (68%)	16 (64%)
N	8 (32%)	8 (32%)	9 (36%)
# of Symptoms/Patient at Baseline (median, range)	4 (3–5)	4 (3–5)	4 (3–5)
Frequency of Symptoms at Baseline			
Fever	25 (100%)	25 (100%)	24 (96%)
Cough	21 (84%)	23 (92%)	20 (80%)
Sore Throat	18 (72%)	19 (76%)	17 (68%)
Body Aches and Pains	9 (36%)	7 (28%)	7 (28%)
Loss of Taste/Smell	6 (24%)	5 (20%)	9 (36%)
Headache	5 (20%)	4 (16%)	5 (20%)
Other	11 (44%)	10 (40%)	14 (56%)
Tiredness	1 (4%)	1 (4%)	0
Shortness of Breath/Difficulty Breathing	0	0	1 (4%)

**Table 2:** WHO Clinical Progression Scale. Shown is the WHO Clinical Progression Scale score by study day and change from baseline.

Visit	Statistics	Placebo	Probenecid 500 mg Bid	Probenecid 1000 mg Bid
Baseline	n	25	25	25
Baseline	Mean	2	2	2
Baseline	SD	0	0	0
Day 1	n	25	25	25
Day 1	Mean	2	2	2
Day 1	SD	0	0	0
Day 3 ± 1	n	25	25	25
Day 3 ± 1	Mean	2	2	2
Day 3 ± 1	SD	0	0	0.2
Change from baseline to Day 3 ± 1	LSmean	0.04	0	0.04
Change from baseline to Day 3 ± 1	95% CI for LSmean	(−0.0251, 0.1051)	(−0.0651, 0.0651)	(−0.0251, 0.1051)
Change from baseline to Day 3 ± 1	*p*-value	0.2247	>0.9999	0.2247
Day 5 ± 1	n	25	25	25
Day 5 ± 1	Mean	1.8	2	1.7
Day 5 ± 1	SD	0.37	0.2	0.56
Change from baseline to Day 5 ± 1	LSmean	0.28	−0.12	0.16
Change from baseline to Day 5 ± 1	95% CI for LSmean	(0.0521, 0.5079)	(−0.3479, 0.1079)	(−0.0679, 0.3879)
Change from baseline to Day 5 ± 1	*p*-value	0.0167	0.2973	0.1659
Day 10 ± 1	n	25	25	25
Day 10 ± 1	Mean	1	0.2	0.1
Day 10 ± 1	SD	0.89	0.55	0.4
Change from baseline to Day 10 ± 1	LSmean	0.08	0.88	0.96
Change from baseline to Day 10 ± 1	95% CI for LSmean	(−0.2849, 0.4449)	(0.5151, 1.2449)	(0.5951, 1.3249)
Change from baseline to Day 10 ± 1	*p*-value	0.6634	<0.0001	<0.0001
Day 15 ± 1	n	25	25	25
Day 15 ± 1	Mean	0.1	0	0
Day 15 ± 1	SD	0.4	0	0
Change from baseline to Day 15 ± 1	LSmean	0	0.08	0.08
Change from baseline to Day 15 ± 1	95% CI for LSmean	(−0.1302, 0.1302)	(−0.0502, 0.2102)	(−0.0502, 0.2102)
Change from baseline to Day 15 ± 1	*p*-value	>0.9999	0.2247	0.2247
Day 28 ± 3	n	25	25	25
Day 28 ± 3	Mean	0	0	0
Day 28 ± 3	SD	0	0	0
Change from baseline to Day 28 ± 3	LSmean	0	0	0
Change from baseline to Day 28 ± 3	95% CI for LSmean	(., .)	(., .)	(., .)
Change from baseline to Day 28 ± 3	*p*-value	<0.0001	<0.0001	<0.0001

LS-mean and *p*-value mentioned in 500 mg bid is for comparison between 500 mg bid vs. Placebo; LS-mean and *p*-value mentioned in 1000 mg bid is for comparison between 1000 mg bid vs. Placebo; LS-mean and *p*-value mentioned in Placebo is for comparison between 500 mg bid vs. 1000 mg bid.

**Table 3:** Adverse events.

System Organ Class Preferred Term	Statistics	Placebo (*N* = 25)	Probenecid/500 mg Bid (*N* = 25)	Probenecid/1000 mg Bid (*N* = 25)	Overall (*N* = 75)
GASTROINTESTINAL DISORDERS	n (%) E	2 (8.0) 2	2 (8.0) 2	3 (12.0) 5	7 (9.3) 9
CONSTIPATION	n (%) E	1 (4.0) 1	1 (4.0) 1	1 (4.0) 1	3 (4.0) 3
HYPERCHLORHYDRIA	n (%) E	1 (4.0) 1	1 (4.0) 1	0 (0.0) 0	2 (2.7) 2
NAUSEA	n (%) E	0 (0.0) 0	0 (0.0) 0	2 (8.0) 2	2 (2.7) 2
VOMITING	n (%) E	0 (0.0) 0	0 (0.0) 0	2 (8.0) 2	2 (2.7) 2
GENERAL DISORDERS & ADMINISTRATION SITE CONDITIONS	n (%) E	1 (4.0) 1	0 (0.0) 0	0 (0.0) 0	1 (1.3) 1
ASTHENIA	n (%) E	1 (4.0) 1	0 (0.0) 0	0 (0.0) 0	1 (1.3) 1
NERVOUS SYSTEM DISORDERS	n (%) E	0 (0.0) 0	1 (4.0) 1	0 (0.0) 0	1 (1.3) 1
HEADACHE	n (%) E	0 (0.0) 0	1 (4.0) 1	0 (0.0) 0	1 (1.3) 1

E: number of events; N: number of subjects dosed with each treatment; n: number of subjects with the adverse event and particular category.

## Data Availability

The data that support the findings of this study are available from the corresponding author upon reasonable request.

## References

[1] Wu Y.C., Chen C.S., Chan Y.J. (2020). The outbreak of COVID-19: An overview. J. Chin. Med. Assoc..

[2] WHO Coronavirus (COVID-19) Dashboard/WHO Coronavirus (COVID-19) Dashboard with Vaccination Data. https://covid19.who.int/?mapFilter=deaths.

[3] Ponnampalli S., Venkata Suryanarayana Birudukota N., Kamal A. (2022). COVID-19: Vaccines and therapeutics. Bioorg. Med. Chem. Lett..

[4] FDA Announces Bebtelovimab Is Not Currently Authorized in Any US Region. https://www.fda.gov/drugs/drug-safety-and-availability/fda-announces-bebtelovimab-not-currently-authorized-any-us-region.

[5] Ying B., Whitener B., VanBlargan L.A., Hassan A.O., Shrihari S., Liang C.Y., Karl C.E., Mackin S., Chen R.E., Kafai N.M. (2022). Protective activity of mRNA vaccines against ancestral and variant SARS-CoV-2 strains. Sci. Transl. Med..

[6] Iketani S., Mohri H., Culbertson B., Hong S.J., Duan Y., Luck M.I., Annavajhala M.K., Guo Y., Sheng Z., Uhlemann A.-C. (2023). Multiple pathways for SARS-CoV-2 resistance to nirmatrelvir. Nature.

[7] Collins P.L., Fearns R., Graham B.S., Anderson L., Graham B. (2013). Respiratory Syncytial Virus: Virology, Reverse Genetics, and Pathogenesis of Disease. Challenges and Opportunities for Respiratory Syncytial Virus Vaccines. Current Topics in Microbiology and Immunology.

[8] Perwitasari O., Yan X., Johnson S., White C., Brooks P., Tompkins S.M., Tripp R.A. (2013). Targeting organic anion transporter 3 with probenecid as a novel anti-influenza a virus strategy. Antimicrob. Agents Chemother..

[9] Murray J., Hogan R.J., Martin D.E., Blahunka K., Sancilio F.D., Balyan R., Lovern M., Still R., Tripp R.A. (2021). Probenecid potently inhibits SARS-CoV-2 replication in vivo and in vitro. Sci. Rep..

[10] Rosli S., Kirby F.J., Lawlor K.E., Rainczuk K., Drummond G.R., Mansell A., Tate M.D. (2019). Repurposing drugs targeting the P2X7 receptor to limit hyperinflammation and disease during influenza virus infection. Br. J. Pharmacol..

[11] Silverman W., Locovei S., Dahl G. (2008). Probenecid, a gout remedy, inhibits pannexin 1 channels. Am. J. Physiol. Cell Physiol..

[12] Weaver A.K., Head J.R., Gould C.F., Carlton E.J., Remais J.V. (2022). Environmental Factors Influencing COVID-19 Incidence and Severity. Annu. Rev. Public Health.

[13] Medina C., Chiu Y., Stremska M., Lucas C., Poon I., Tung K., Elliott M., Desai B., Lorenz U., Bayliss D. (2021). Pannexin 1 channels facilitate communication between T cells to restrict the severity of airway inflammation. Immunity.

[14] Beyer R.H., Wiebelhaus V.D., Russe H.F., Peck H.M., McKinney S.E. (1950). Benemid: An anticatabolite; Its pharmacological properties. Fed. Proc..

[15] Boger W.P., Strickland S.C. (1955). Probenecid (Benemid): Its uses and side effects in 2502 Patients. Am. Med. Assoc. Arch. Intern. Med..

[16] Robbins N., Koch S.E., Tranter M., Rubinstein J. (2012). The history and future of probenecid. Cardiovasc. Toxicol..

[17] WHO Working Group on the Clinical Characterisation and Management of COVID-19 infection (2020). A minimal common outcome measure set for COVID-19 clinical research. Lancet Infect Dis..

[18] WHO (2020). COVID-19 Case Definitions. https://apps.who.int/iris/rest/bitstreams/1322790/retrieve.

[19] PROBENECID-Probenecid Tablet, Film Coated Package Insert. Lannett Company, Inc. Revision 1/2021. https://dailymed.nlm.nih.gov/dailymed/fda/fdaDrugXsl.cfm?setid=ab497fd8-00c3-4364-b003-b39d21fbdf38&type=display.

[20] Tripp R.A., Martin D.E. (2022). Repurposing Probenecid to Inhibit SARS-CoV-2, Influenza Virus, and Respiratory Syncytial Virus (RSV) Replication. Viruses.

[21] Caly L., Li H.M., Bogoyevitch M.A., Jans D.A. (2017). c-Jun N-terminal kinase activity is required for efficient respiratory syncytial virus production. Biochem. Biophys. Res. Commun..

[22] Feng Z., Xu L., Xie Z. (2022). Receptors for Respiratory Syncytial Virus Infection and Host Factors Regulating the Life Cycle of Respiratory Syncytial Virus. Front. Cell Infect. Microbiol..

[23] Cheng M.H., Kim S.J. (2020). Inhibitory Effect of Probenecid on Osteoclast Formation via JNK.; ROS and COX-2. Biomol. Ther..

[24] Zeke A., Misheva M., Reményi A., Bogoyevitch M.A. (2016). JNK Signaling: Regulation and Functions Based on Complex Protein-Protein Partnerships. Microbiol. Mol. Biol. Rev..

[25] Kumar R., Khandelwal N., Thachamvally R., Tripathi B.N., Barua S., Kashyap S.K., Maherchandani S., Kumar N. (2018). Role of MAPK/MNK1 signaling in virus replication. Virus Res..

[26] Sehgal V., Ram P.T. (2013). Network Motifs in JNK Signaling. Genes Cancer.

[27] Martovetsky G., Tee J.B., Nigam S.K. (2013). Hepatocyte nuclear factors 4α and 1α regulate kidney developmental expression of drug-metabolizing enzymes and drug transporters. Mol. Pharmacol..

[28] Karyakarte R.P., Das R., Taji N., Yanamandra S., Shende S., Joshi S., Karekar B., Bawale R., Tiwari R., Jadhav M. (2022). An Early and Preliminary Assessment of the Clinical Severity of the Emerging SARS-CoV-2 Omicron Variants in Maharashtra, India. Cureus.

